# Association between C-reactive protein and hemoglobin in US rheumatoid arthritis patients based on NHANES data analysis

**DOI:** 10.1038/s41598-025-93720-z

**Published:** 2025-03-14

**Authors:** Defang Liu, Jiao Yan, Ting Luo, Ling Yang

**Affiliations:** Department of Traditional Chinese Medicine, The General Hospital of Western Theater Command, No. 270 Tianhui Road, Chengdu, 610083 Sichuan China

**Keywords:** Rheumatoid arthritis, C-reactive protein, Hemoglobin, All-cause mortality, Covariates, NHANES, Immunology, Rheumatology, Risk factors

## Abstract

The poor prognosis of rheumatoid arthritis (RA) and its potential for complications highlight the importance of understanding factors that are associated with incidence and mortality rates. The inclusion criteria of this study were RA-related data from 1999 to 2018 in the National Health and Nutrition Examination Survey (NHANES) dataset. Based on certain screening criteria, a total of 610 subjects were recruited. The Low CRP group (< 3 mg/L) and the High CRP group (> 3 mg/L) were significantly different in gender, poverty-to-income ratio, body mass index, hypertension, hemoglobin (Hb), hematocrit, and mean corpuscular hemoglobin. KM survival result revealed that male RA patients in the Low Hb group had a significantly lower survival rate than those in the High Hb group (*P* < 0.0001), while female RA patients in the Low Hb group showed no statistically significant difference compared with the High Hb group (*P* = 0.13). Importantly, there was a nonlinear relationship between Hb and all-cause mortality in RA patients. In this study, we identified Hb as a protective factor against CRP in RA patients and also observed its association with the prognosis of RA. Consequently, regulating Hb levels might be considered to be associated with the progression of RA.

## Introduction

Rheumatoid arthritis (RA) is a prototypical systemic autoimmune disease that causes joint inflammation and progressive disability^1^. The global prevalence of RA is as high as 1%, resulting in premature death and considerable socioeconomic costs^2^. Awareness and the ability to treat and control autoimmune inflammation in patients with RA have increased over time, but the condition remains incurable for most patients. Patients with RA suffer from a variety of extra-articular manifestations and complications related to their persistent inflammatory state^3^. The most common extra-articular manifestation is anemia, affecting approximately half of RA patients^4^.

Anemia is defined as a lower-than-normal level of hemoglobin (Hb)^5^. C-reactive protein (CRP) is a commonly used marker of inflammation and RA activity^6^. However, there are few research reports on the relationship between Hb and CRP levels in RA patients or its association with mortality; no in-depth statistical analysis of this association has been conducted. This study therefore explored the relationship between Hb and CRP and its association with mortality in RA patients, based on relevant data in the National Health and Nutrition Examination Survey (NHANES) database.

## Materials and methods

### Data and participants

**Fig. 1:**
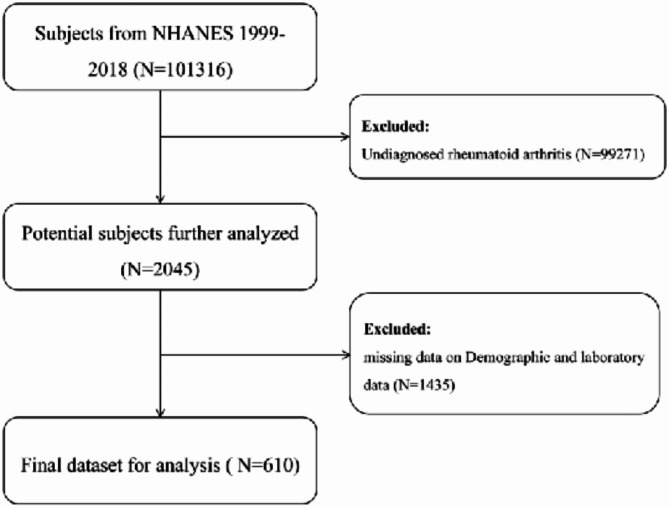
Flow chart of the sample collection in this study.

NHANES database (https://www.cdc.gov/nchs/nhanes/index.htm) was applied to access the data used in this study, which was an annual study of the health of the U.S. population. According to the Declaration of Helsinki, every participant has provided written informed consent. Inclusion criteria for this study included all subjects from 1999 through 2018, excluding those who were < 18 years of age, pregnant women, and individuals who lacked clinically important data. A final total of 610 subjects were recruited for the follow-up study (Fig. 1).

### Definition of main variables

The diagnosis of RA was established based on self-reported data obtained from the Medical Status Questionnaire (MCQ). Specifically, according to the question in MCQ160A, subjects who were informed by doctors or other health professionals that they had arthritis were asked to confirm the type of arthritis using MCQ195. Those who indicated RA were included in this study.

The main outcome was all-cause mortality. This study utilized the data from the NHANES Public Use Associated Mortality Document, which was linked by the National Center for Health Statistics (NCHS) to the National Mortality Index and followed up until December 31, 2015. The case definition of potential cause of death was based on the International Classification of Diseases, Tenth Revision (ICD-10).

### Evaluation of covariates

In this study, a number of important covariates were included in order to assess the association of these covariates, which contained age (DMDHRAGE), gender (RIAGENDR), race (RIDRETH1), marriage status (DMDHRMAR), education (DMDEDUC2, AUQ440), poverty-to-income ratio (PIR) (INDFMPIR), body mass index (BMI) (BMXBMI), smoking (SMQ040), drinking (ALQ101), hypertension (BPQ020), diabetes (DIQ010), complete blood count (LB2WBCSI, LB2RBCSI), health insurance (HIQ011), Hb (MCHC), CRP (LBXCRP), and Albumin (LBDSALSI).

Among these covariates, the race encompassed Mexican Americans, non-Hispanic blacks, non-Hispanic whites, other Hispanics, and individuals of other races-including Multi-Racial. Marital status was categorized as married, never married, or widowed/divorced/separated. Education included completion of grades 9–11, college graduation or above, high school graduation or equivalent, less than 9th grade, and some college or AA degree. Smoking habits were classified into three categories: daily smokers (every day), non-smokers (not at all), and occasional smokers (some days). The complete blood count analysis comprised measurements of white blood cell count, red blood cell count, hematocrit (HCT), mean corpuscular hemoglobin (MCH), and red cell distribution width (RDW). The remaining covariates were continuous variables.

### Statistical analysis

The mean [standard deviation (SD)] for continuous variables and the number (%) for categorical variables were used to summarize the baseline characteristics. Baseline table was generated using tableone package (v 0.13.2)^7^. Given the variations in the definitions of “normal” levels of hemoglobin and CRP among different laboratories, we adopted the median value as the standard criterion for differentiating patients into high and low CRP groups. The chi-square test was utilized to evaluate categorical variables, while Student’s t-test was employed to compare continuous variables between these two groups^8^. The sequential multifactorial generalized linear models (GLM) were generated utilizing the nhanesA package (v 1.0.2)^9^ to reveal the correlation between Hb and CRP subgroups in RA patients. To be specific, Model 1 was unadjusted, while Model 2 additionally adjusted for age, gender, and race. Model 3 further adjusted for other covariates. Meanwhile, correlations between covariates and CRP risk in different populations were analyzed using weighted logistic regression to confirm their stability. Afterwards, Kaplan–Meier (K-M) analysis was implemented via survival package (v 3.5.3)^10^ to explore the relationship between Hb levels and prognosis in RA patients. Additionally, restricted cubic spline (RCS) analysis was conducted with the rms package (v 6.5.0)^11^ to examine whether there existed a linear or nonlinear association between Hb levels and all-cause mortality. Bioinformatic analysis was implemented in R program (v 4.2.2) (*P* < 0.05).

## Results

### Baseline statistics of subjects

The association of variables with CRP was evaluated using the student’s t-test and the chi-square test. The RA sample was split into two groups: the Low CRP group (n = 285) with CRP levels < 3 mg/L and the High CRP group (n = 325) with CRP levels > 3 mg/L. The findings showed that there were significant differences in gender, PIR, BMI, hypertension, Hb, HCT, MCH, RDW, and albumin between the High and Low CRP groups (*P* < 0.05) (Table 1).

**Table 1:** Analysis of baseline characteristics of RA according to CRP levels.

	Low CRP N1 = 285	High CRP N2 = 325	*P* value
Age (years, mean (SD))	59.77 (14.73)	59.59 (14.39)	0.876
Gender (Male (%))	166 (58.2)	154 (47.4)	0.006
Race (%)			0.464
Mexican American	39 (13.7)	49 (15.1)	
Non-Hispanic Black	65 (22.8)	90 (27.7)	
Non-Hispanic White	155 (54.4)	161 (49.5)	
Other Hispanic	19 (6.7)	21 (6.5)	
Other Race-Including Multi-Racial	7 (2.5)	4 (1.2)	
Marriage status (%)			0.304
Married	177 (62.1)	182 (56.0)	
Never married	20 (7.0)	25 (7.7)	
Widowed/Divorced/Separated	88 (30.9)	118 (36.3)	
Education (%)			0.075
9–11th grade	57 (20.0)	87 (26.8)	
College graduate or above	24 (8.4)	17 (5.2)	
High school graduate or equivalent	74 (26.0)	78 (24.0)	
Less than 9th grade	45 (15.8)	64 (19.7)	
Some college or AA degree	85 (29.8)	79 (24.3)	
PIR (mean (SD))	2.23 (1.51)	2.01 (1.43)	0.078
BMI (mean (SD))	28.24(6.60)	31.28 (7.51)	< 0.001
Smoking (%)			0.5
Every day	105 (36.8)	105 (32.3)	
Not at all	168 (58.9)	205 (63.1)	
Some days	12 (4.2)	15 ( 4.6)	
Drinking (%)	208 (76.2)	227 (72.1)	0.297
Hypertension (%)	148 (51.9)	192 (59.4)	0.075
Diabetes (%)	58(20.4)	80 (24.6)	0.313
Health insurance (%)	244(85.6)	285 (88.0)	0.427
White blood cell count (1000 cells/uL, mean (SD))	7.11 (2.17)	8.11 (2.77)	< 0.001
Red blood cell count (million cells/uL, mean (SD))	4.60 (0.48)	4.58 (0.51)	0.567
Hb (g/dL, mean (SD))	14.42 (1.51)	13.90 (1.57)	< 0.001
HCT (%, mean (SD))	42.26 (4.30)	41.03 (4.55)	0.001
MCH (pg, mean (SD))	31.43 (2.11)	30.51 (2.80)	< 0.001
RDW (%, mean (SD))	13.01 (1.22)	13.62 (2.13)	< 0.001
Albumin (g/L, mean (SD))	42.74 (3.10)	40.83 (3.60)	< 0.001

### The relationship between CRP and Hb

**Fig. 2:**
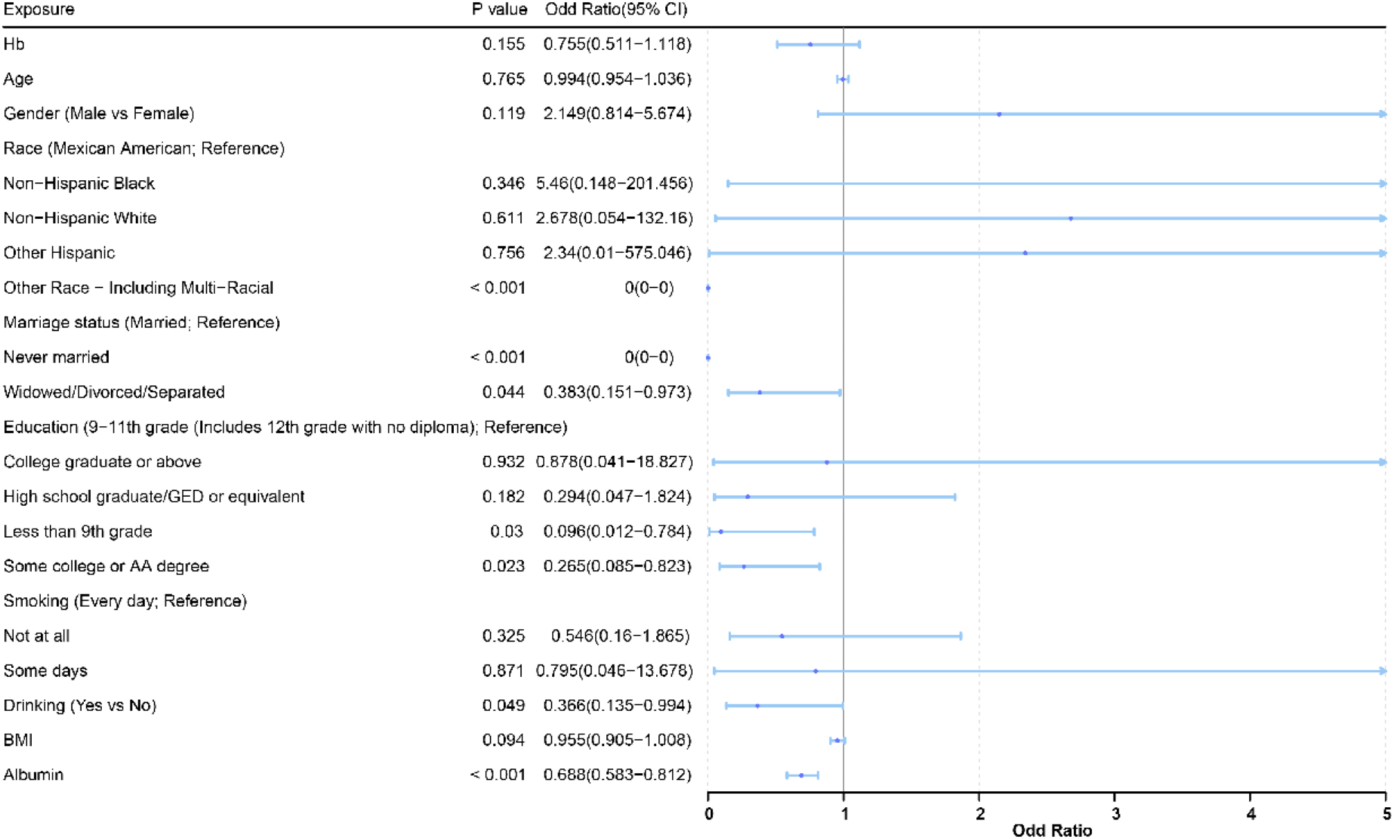
Multivariable cox proportional hazard ratios for RA with HB and CRP in subgroups stratified by patients’ characteristics.

The sequential multifactorial GLM was constructed in order to better investigate the link between Hb and CRP. There was a significant correlation between Hb and CRP in both models 1 (odds ratio (OR) = 0.791; 95% confidence interval [CI] = 0.699–0.895, *P* = 0.000364) and model 2 (OR = 0.84; 95% CI = 0.737–0.958, *P* = 0.0102), with Hb showing a protective effect on CRP levels. Even after a thorough adjustment for variables in model 3, this significant link remained statistically significant (OR = 0.821; 95% CI = 0.713–0.946, *P* = 0.00749), indicating that other factors did not significantly confuse the effect of Hb on CRP (Table 2). Additionally, stratified analyses indicated that there was still a significant association between Hb and CRP in RA (Fig. 2).

**Table 2:** Analysis of the association between exposure factors and risk of RA.

Characteristic	Model 1		Model 2		Model 3	
	OR (95% CI)	*P* value	OR (95% CI)	*P* value	OR (95% CI)	*P* value
Hb	0.791 (0.699–0.895)	0.000364	0.84 (0.737–0.958)	0.0102	0.821 (0.713–0.946)	0.00749

### Correlation between Hb levels and all-cause mortality in patients with RA

**Fig. 3:**
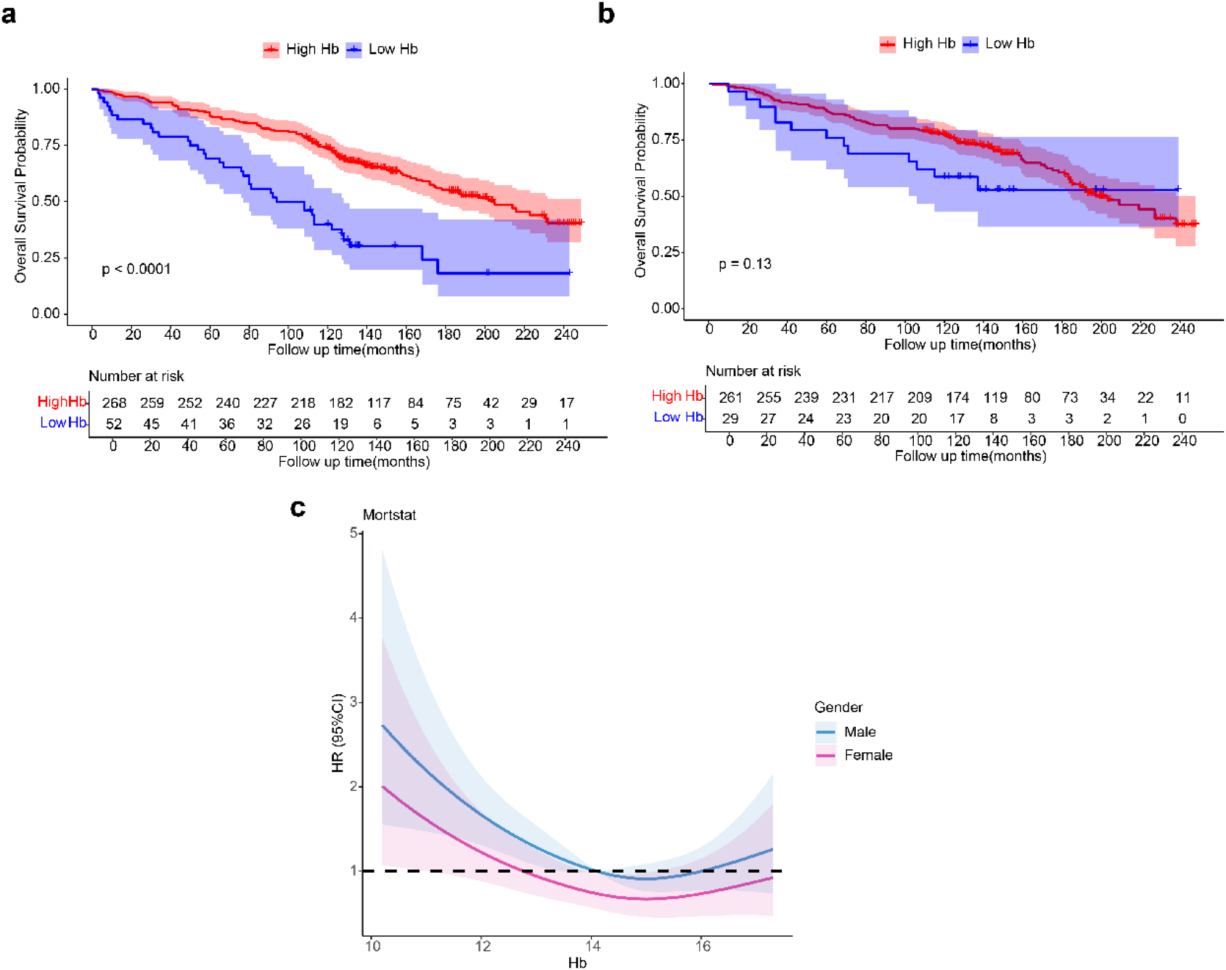
Effect of HB levels on survival differences and all-cause mortality in RA. (**a**) Survival differences in RA (male) according to HB levels. (**b**) Survival differences in RA (female) according to HB levels. (**c**). Relationship between HB and all-cause mortality in RA. The blue line represents the trend of HR with changes in Hb levels in male subjects, showing a protective effect when Hb is between 14–16 g/dL, and a risk factor outside this range. The pink line represents the trend of HR with changes in Hb levels in female subjects, showing a protective effect when Hb is between 13 and 17 g/dL. The result from the linear assessment yielded *P* = 0.0005, suggesting that Hb is nonlinearly related to mortality.

Based on data from the World Health Organization (WHO), male subjects were classified into Low Hb group (Hb < 13 g/dL) and High Hb group (Hb > 13 g/dL), while female subjects were classified into Low Hb group (Hb < 12 g/dL) and High Hb group (Hb > 12 g/dL)^12^. The link between Hb and all-cause mortality in RA patients was then determined by analyzing the survival difference between the two groups using K-M survival analysis. According to the findings, the survival outcomes of RA male patients with low hemoglobin levels were significantly worse, with a significant difference (*P* < 0.0001), while the survival outcomes of RA female patients with low hemoglobin levels were worse, but no significant difference was found (*P* = 0.13) (Fig. 3a, b). After that, RCS analysis revealed that the relationship between Hb and all-cause mortality is not linear, but nonlinear, meaning that the relationship between the two may exhibit a complex pattern as Hb levels change (*P* = 0.0005). Specifically, for male patients with RA, a protective effect was seen in the range of approximately 14–16 g/dL, while outside this range it became a risk factor. Conversely, in female RA patients, Hb acted as a protective factor within the interval of about 13–17 g/dL. Moreover, both genders demonstrated a threshold at around 15 g/dL; above this level, higher Hb values were associated with smaller hazard ratios (HR), whereas below this threshold, elevated Hb levels corresponded to larger HR (Fig. 3c).

## Discussion

Common types of anemia in RA patients include anemia of chronic disease, drug-induced immune hemolytic anemia (DIIHA), and iron deficiency anemia (IDA); further forms are associated with comorbid hematological diseases^13^. In this study of 610 US RA patients, we found a statistically significant association between Hb and CRP that persisted after adjustment for demographic characteristics, body mass index, smoking, and drinking status.

Previous studies have focused on the effect of inflammation on anemia. In inflammatory states, cytokine interleukin-6 (IL-6) stimulates the production of hepcidin by hepatocytes, which binds to its receptor and suppresses the release and absorption of iron; less iron is subsequently available for the production of Hb in hematopoietic tissues^14–16^. Simultaneously, inflammatory cytokines such as Interleukin-1β (IL-1β) and tumor necrosis factor-α (TNF-α) inhibit the production and release of erythropoietin (EPO) from the kidneys, further contributing to the anemic state^14^. Patients with anemia and RA have been reported to be associated with higher disease activity and structural damage^17^, suggesting a link between Hb levels and RA progression. This is further supported by evidence that anti-inflammatory therapies can alleviate anemia symptoms in RA patients^4^. Under inflammatory conditions, the liver modulates its protein synthesis profile, reducing albumin production and increasing the synthesis of CRP^18^. During the highly active RA, the levels of inflammatory cytokines such as IL-6, IL-1β, and TNF-α increase^19^. IL-6 inhibits albumin gene transcription in hepatocytes through signal transducer and activator of transcription 3 (STAT3) activation and indirectly impairs serum albumin synthesis by altering hepatic metabolism^20^. Concurrently, IL-6, IL-1β, and TNF-α promote the production of CRP by the liver, adipocytes, leukocytes, and lymphocytes^21^, leading to an increase in CRP levels and a decrease in albumin levels during the highly active RA. In this study, the High CRP group exhibited significantly lower albumin levels compared to the Low CRP group (*P* < 0.05), consistent with this phenomenon. CRP is a key marker of inflammation, while the albumin level decreases not only in inflammatory conditions but also due to factors such as liver diseases, kidney diseases, and malnutrition^22^. Therefore, CRP was selected as the primary biomarker for group stratification in this study.

The role of Hb in inhibiting inflammation is rarely discussed. As the primary constituent of erythrocytes, Hb is responsible for transporting oxygen and carbon dioxide in the bloodstream; it is the most widely distributed heme protein in nature, existing in almost all vertebrates and some invertebrates^23,24^. The Hb molecule is composed of four polypeptide chains, each containing a heme fragment. Hb serves various functions including drug delivery, sulfide transport, acid–base balance and osmotic pressure regulation, and peroxidase and antibacterial activity^25–28^. The scavenger receptor CD163, which takes part in the recycling of the heme iron, is expressed by macrophages and circulating monocytes^29,30^ . Based on its clearance mechanisms, Hb is a promising potential drug delivery agent, especially for macrophage-associated diseases. Macrophages are immune cells involved in both non-specific (innate immunity) and specific defense (cellular immunity); they play an important role in the occurrence, development, and prognosis of autoimmune diseases such as RA. Macrophages are mainly divided into M1 and M2 types. In early RA, M1 macrophages activate T-cells through antigen presentation and secrete several inflammatory cytokines, including IL-1β, IL-6, and TNF-α. They also recruit more immune cells and activate fibroblast-like synovium cells that further promote inflammatory responses. In the later stages of RA inflammation resolution, M2 macrophages play a role in inhibiting inflammation^31,32^. Hb could therefore be recruited in the distribution of natural products, drugs, or any other materials that target macrophages. Hb can stimulate macrophages to produce a variety of cytokines which have anti-inflammatory and immunomodulatory effects^33^. On the other hand, under physiological conditions, Hb can use its peroxidase activity to catalyze the production of appropriate amounts of reactive oxygen species (ROS), promoting immunity, repair, survival, growth, and other beneficial mechanisms^34,35^. Under pathological conditions, Hb promotes the production of high concentrations of ROS, which are implicated in many diseases, including malignant tumors, diabetes, and arthritis^36,37^. In summary, the decrease of Hb in patients with RA will promote inflammation, which in turn further reduces Hb levels, forming a vicious circle and ultimately aggravating the disease. CRP is a general marker of inflammation, which increases in the active stage of RA^38,39^. In this study, we identified Hb as a protective factor for CRP in patients with RA and a key factor influencing their prognosis. The survival rate of RA patients with low levels of Hb was significantly lower.

Based on the statistical results of this study, the average BMI of RA patients with the Low CRP group (28.24 ± 6.60 kg/m^2^) and those with the High CRP group (31.28 ± 7.51 kg/m^2^) both met the US definition of overweight (BMI of 25 to < 30 kg/m^2^) (*P* < 0.05). Our findings suggest that a substantial number of RA patients, particularly those with high CRP levels during the highly active and progressive RA, are troubled by overweight, which is generally consistent with previous research^40^. The reciprocal influence between BMI and RA is intricate and multifaceted. RA patients, plagued by long-term joint damage and pain, have restricted daily activities and a significant reduction in physical activity, thus increasing their risk of overweight^41^. Meanwhile, adipose tissue continuously secretes pro-inflammatory cytokines, which not only severely damage bones and cartilage^40^, but may also extend to reduced muscle mass (cachexia), further exacerbating RA disease activity^42^. Clinical studies indicate that patients with RA and obesity exhibit worse clinical symptoms and are more difficult to achieve remission compared to non-obese counterparts^43^. Common RA medications, including corticosteroids^44^ and TNF inhibitors^45^, are associated with potential weight gain. Furthermore, obesity can reduce the effectiveness of TNF inhibitors in treating RA^46^. This also, to some extent, explains why the BMI of RA patients with high CRP in this study is significantly higher than that of RA patients with low CRP.

In this study, we ascertained that Hb levels (14–16 g/dL in males; 13–17 g/dL in females) serve as protective factors for RA patients and are pivotal determinants in dictating their prognoses. However, there are a pronounced association between sex differences and Hb levels on the survival outcomes of RA patients. Concretely, male RA patients of Low Hb group experience significantly worse survival outcomes (*P* < 0.0001), while the female patients of Low Hb group, although having relatively poor survival outcomes, show no statistically significant difference (*P* = 0.13). The complex sex differences in survival outcomes stem from multiple interacting mechanisms. Hormonal discrepancies assume a pivotal role. In males, the lack of estrogen-mediated anti-inflammatory and cardiovascular-protective effects subjects them to intense inflammatory reactions and elevated cardiovascular risks^47^. The impaired oxygen transport caused by low Hb exacerbates cardiac stress, thereby further increasing the risk of cardiovascular incidences such as heart failure and myocardial infarction^48^. Conversely, females’ long-term physiological processes, such as menstruation, endow their hematopoietic systems with unique adaptability and compensatory capacity^49^. Additionally, women are much more likely than men to seek medical care^50^, potentially alleviating disease impacts on survival. Among RA patients, these findings indicate that low hemoglobin levels may serve as a crucial prognostic indicator for poor outcomes in male patients, necessitating intensified anemia screening and management. In female patients, although the effects lack statistical significance, the influence of anemia on their quality of life nonetheless merits attention.

Our study had several limitations. First, RA was ascertained by self-report, potentially leading to overreporting, as this study evidenced by the 2.02% self-reported RA prevalence in NHANES (2045 out of 101,316 participants), significantly higher than the 0.41–0.54% prevalence in US administrative insurance claims databases (2004–2014)^51^ and the 0.46% estimate from the 2021 Global Burden of Disease (GBD) study^52^. This may due to differences in data collection methods: NHANES depends on self-reported surveys, while the other two rely on ICD codes; NHANES uses random sampling for the general US population, and administrative databases focus on commercially insured individuals, and the GBD database uses multiple sources with estimation and modeling. These methodological variations highlight inconsistencies in RA prevalence across datasets, which may overemphasize RA risk or prognostic factors. Second, due to the nature of the cross-sectional study design of the NHANES, causality cannot be inferred between Hb and CRP levels. Finally, although the multiple regression models included a broad set of additional factors as covariates, not all plausible confounding factors were available for inclusion in the models.

## Conclusion

The results of our study estimated Hb as a protective factor against CRP in RA patients. Therefore, regulating Hb levels might be a potential strategy for reducing CRP levels and managing the progression of RA.

## Data Availability

The original contributions presented in the study are included in the article, further inquiries can be directed to the corresponding authors.
